# Reaching every child with rotavirus vaccine: Report from the 10th African rotavirus symposium held in Bamako, Mali

**DOI:** 10.1016/j.vaccine.2017.08.084

**Published:** 2017-10-09

**Authors:** Samba O. Sow, A. Duncan Steele, Jason M. Mwenda, George E. Armah, Kathleen M. Neuzil

**Affiliations:** a Centre pour le Développement des Vaccins, Bamako, Mali; b Enteric and Diarrheal Diseases, Global Health, Bill & Melinda Gates Foundation, Seattle, WA, USA; c World Health Organization Regional Office for Africa, Brazzaville, People’s Republic of Congo; d Noguchi Memorial Institute for Medical Research, College of Health Sciences, University of Ghana, Legon, Ghana; e Center for Vaccine Development and Institute for Global Health, University of Maryland School of Medicine, Baltimore, MD, USA

**Keywords:** Rotavirus, Vaccine, Africa, Surveillance, Intussusception, Vaccine effectiveness

## Abstract

The Center for Vaccine Development – Mali (CVD – Mali), the World Health Organization’s regional office in Africa (WHO/AFRO), and the CVD at the University of Maryland School of Medicine hosted the 10th African Rotavirus Symposium in Bamako, Mali on 1–2 June 2016. The symposium is coordinated by WHO/AFRO, the Regional Rotavirus Reference Laboratories, and the African Rotavirus Network (ARN), with support from the Bill & Melinda Gates Foundation. The event brings together leading rotavirus researchers, scientists, and policy-makers from across Africa and the world. Over 150 participants, from 31 countries, including 27 in Africa, joined forces to address the theme “Reaching Every Child in Africa with Rotavirus Vaccines.” This symposium, the first in francophone Africa, occurred at an unprecedented time when 33 African countries had introduced rotavirus vaccines into their national immunization programs. The symposium concluded with a Call to Action to introduce rotavirus vaccines in the 21 remaining African countries, to increase access in countries with existing vaccination programs, and to continue surveillance and research on rotavirus and other diarrheal diseases.

## Highlights

• 10th African Rotavirus Symposium, Bamako, Mali, 1–2 June 2016.
• Reaching every child in Africa with rotavirus vaccines.
• Global gathering of rotavirus researchers, scientists, and policy-makers.

## 1 Introduction

Preventing rotavirus infection through vaccination is a critical intervention to reduce morbidity and mortality in young children, particularly in settings without accessible or affordable health care [1]. The African Rotavirus Symposium is a gathering of rotavirus experts that occurs every one to two years and provides a unique venue to discuss the latest research findings and global recommendations, and to share monitoring, surveillance, and vaccine introduction data from across Africa and the globe. This report serves as the proceedings for the symposium.

Due to the accelerated vaccine introduction in Africa and the rapid advances in the field, the 9th African Rotavirus Symposium was held in Maputo, Mozambique in December 2015, one year after the 8th African Rotavirus Symposium [2]. The symposium focused on assessing the role of the regional rotavirus surveillance network in defining rotavirus epidemiology in the pre-vaccine era, and the on-going efforts to assess the impact of vaccines and to monitor adverse events [2].

On 1–2 June 2016, the Center for Vaccine Development (CVD)-Mali and the World Health Organization’s regional office in Africa (WHO/AFRO), hosted the 10th African Rotavirus Symposium in collaboration with the Regional Rotavirus Reference Laboratories, and the African Rotavirus Network (ARN).^1^ The symposium included participants from African Ministries of Health and government agencies; the Regional Reference Laboratories; and other rotavirus researchers, scientists, and policy-makers.

**Fig. 1:**
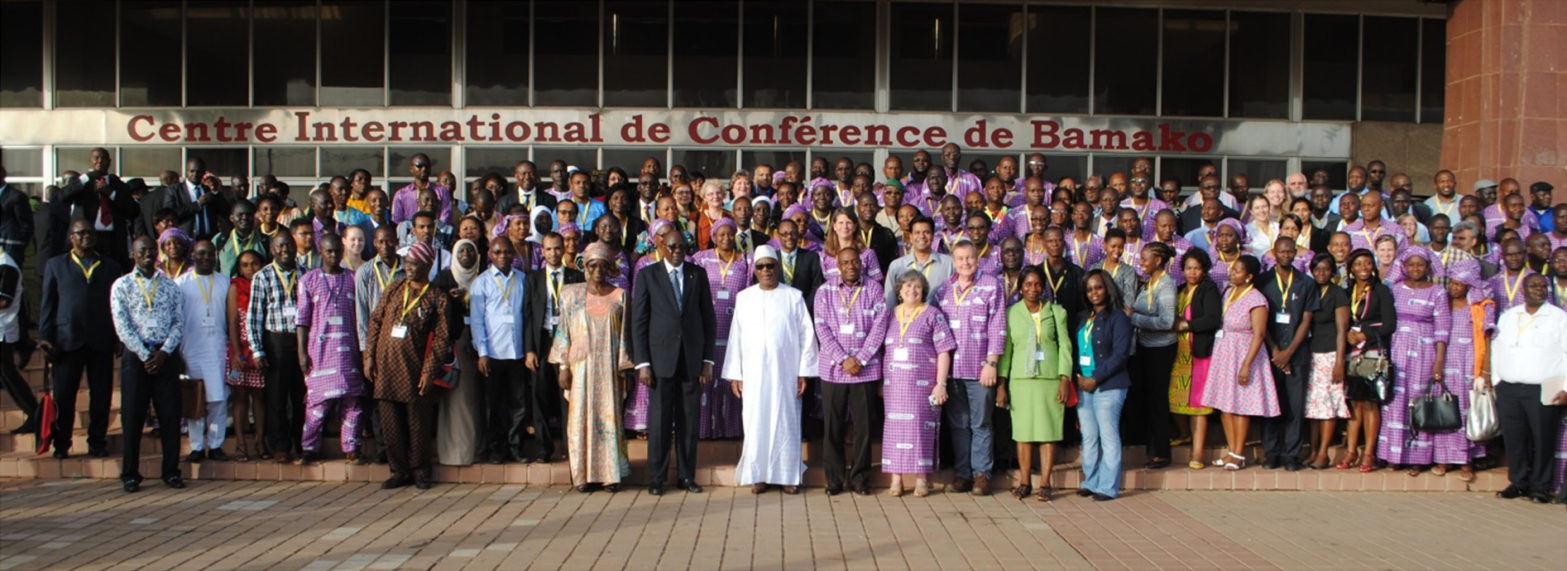
His Excellency Ibrahim Boubacar Keita (center in white), President of the Republic of Mali, with conference attendees at the Opening Ceremony of the 10th African Rotavirus Symposium.

The symposium was officially opened by His Excellency Ibrahim Boubacar Keita, President of the Republic of Mali and Dr. Marie Madeleine Togo, Minister of Health of Mali (Fig. 1). More than 400 dignitaries, including Prime Minister Modibo Keita and other members of the government of Mali, joined symposium participants at the opening ceremony. President Keita welcomed and thanked the conference attendees for their dedication to advancing rotavirus vaccines and improving child health in Mali and throughout Africa.

Over 150 participants from 31 countries – 27 in Africa – attended the symposium, which included invited lectures, oral presentations, panel discussions, and poster presentations. The objectives of the conference, presented by Dr. Jason Mwenda, WHO/AFRO, were to: Share research and findings on global, regional, and country-specific epidemiological trends on rotavirus diarrheal disease; provide updates on vaccine introductions, progress, challenges, and way forward to accelerate vaccine introduction in Africa; share experiences on vaccine impact and safety; and facilitate networking for research, academic, and career growth among researchers, program managers, and policy-makers.

## 2 Proceedings of meeting

### 2.1 Keynote address

Dr. Duncan Steele, Bill & Melinda Gates Foundation, delivered the keynote address entitled “Reaching Every Child with Rotavirus Vaccines.“ Diarrhea is the second leading cause of death in children under five [3], with the highest global mortality rates reported from sub-Saharan Africa [4]. Even for children who survive, rotavirus can have detrimental impacts on nutrition, growth, and well-being [5]. Rotavirus vaccines should be part of a comprehensive strategy to control diarrheal diseases, as recommended by WHO, with the scaling up of both prevention (promotion of early and exclusive breastfeeding, hand washing with soap, and improved water and sanitation) and treatment (including low-osmolarity oral rehydration salts and zinc) [1].

**Fig. 2:**
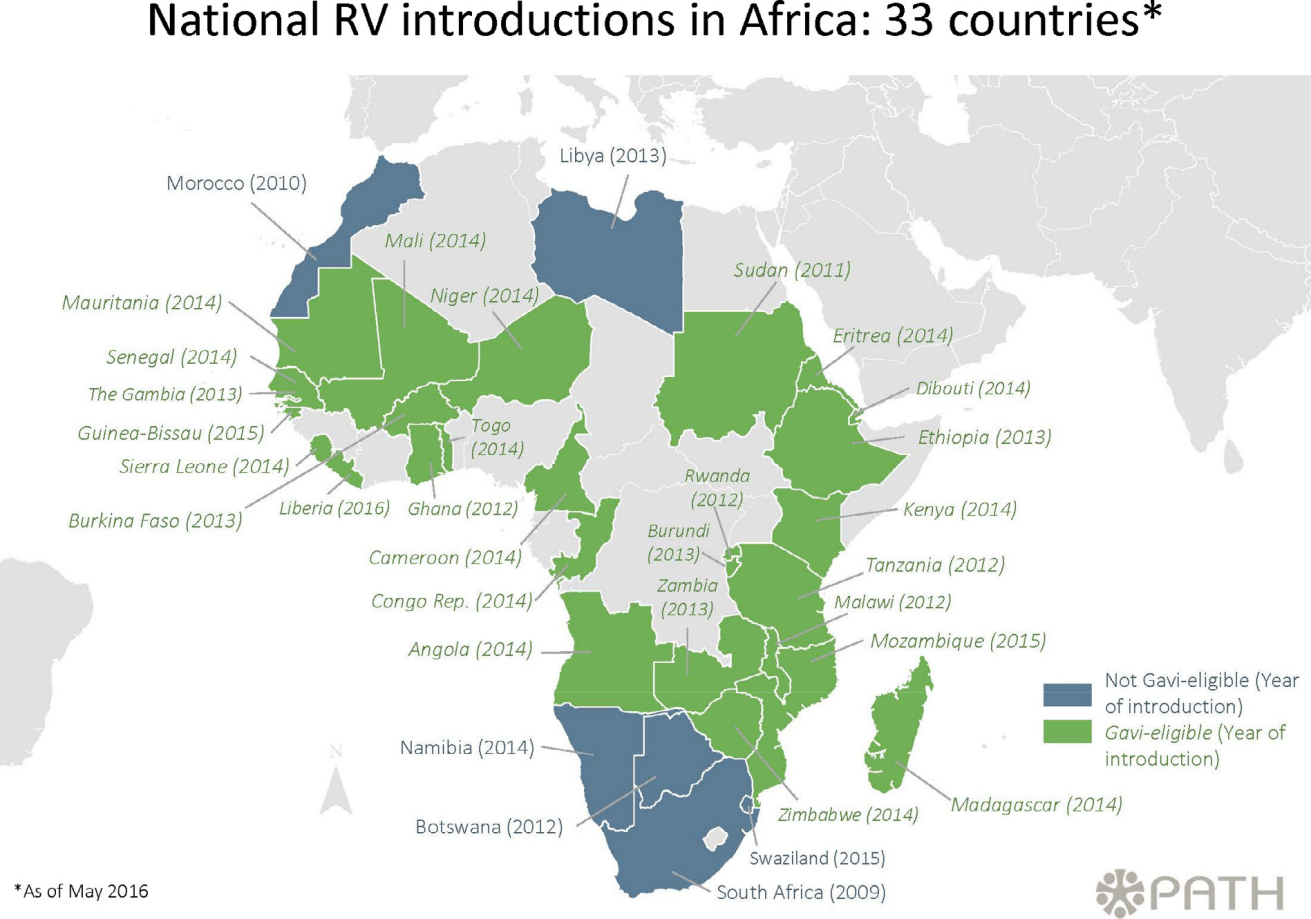
National rotavirus vaccine introductions in Africa. (PATH. 2106. Rotavirus vaccine introductions in Africa. Derived from “Worldwide introductions of rotavirus vaccines by geographic region” available online at: http://sites.path.org/rotavirusvaccine/files/2016/05/PATH-Worldwide-Rotavirus-Vaccine-Introduction-Map-EN-2016.05.01_geo.jpg.)

In 2009, South Africa was the first African country to include rotavirus vaccines in their Expanded Program on Immunization (EPI). As shown in Fig. 2, as of May 2016, 33 African countries (29 in the African Region and 4 in the Eastern Mediterranean Region) have rotavirus vaccine in their EPI, while 21 have yet to introduce the vaccine. Dr. Steele outlined several potential approaches to achieve the goal of reaching every child with rotavirus vaccines. These included enhancing supply by supporting existing and new suppliers, ensuring new rotavirus vaccines have an acceptable presentation, pursuing next generation rotavirus vaccines to improve efficacy, and strengthening the routine immunization system.

Cost and cost-effectiveness are increasingly important factors in country-level decision-making and will become the single biggest challenge to sustaining programs as countries graduate from Gavi, the Vaccine Alliance (Gavi) financing. Dr. Steele presented several cost-effectiveness studies from different African countries and concluded that every scenario explored shows rotavirus vaccines are highly cost-effective [6], [7], [8], [9], [10]. Finally, Dr. Steele challenged participants to ensure equity of access to guarantee vaccines reach children living in communities with the highest rotavirus mortality.

### 2.2 Disease Burden in Africa: rotavirus and beyond

This session summarized rotavirus and norovirus disease burden and the importance of surveillance and analysis for vaccine introduction, monitoring, and policy implications.

Dr. Jacqueline Tate, United States Centers for Disease Control and Prevention (CDC), provided the most recent updates on global rotavirus disease burden. While diarrhea deaths continue to decline, diarrhea remains a leading cause of death among children globally [4]. Rotavirus affects children in both developed and developing countries, however, morbidity and mortality are greatest in resource-poor settings [11].

Based on a literature review and data from the global rotavirus surveillance network coordinated by WHO, Dr. Tate reported the proportion of diarrheal deaths due to rotavirus is declining, but there are disparities related to vaccine access. In 2013, 34 percent of the population in developed countries lived in a post-rotavirus vaccine introduction country compared to less than 10 percent of the population in all other countries. An estimated average of 215,000 (range: 197,000 to 233,000) rotavirus deaths occurred among children less than 5 years of age in 2013. Of these remaining rotavirus deaths, 56 percent are estimated to occur in sub-Saharan Africa.

Dr. Karen Kotloff, CVD, provided data from a re-analysis of the landmark Global Enteric Multicenter Study (GEMS), which assessed incidence, etiology, and adverse clinical consequences of severe diarrhea in children under five in low resource settings, three in sub-Saharan Africa [5]. GEMS identified five pathogens, including rotavirus, that account for half the moderate-to-severe diarrhea cases. The introduction of rotavirus vaccines is expected to impact not only rotavirus-specific morbidity and mortality, but also other adverse outcomes associated with diarrhea and potentially the relative contribution of other pathogens as etiologic agents.

Rotavirus surveillance at the regional and country level in children under five is resource-intensive, yet essential to inform policy decisions, support vaccine introduction, and monitor programs.^2^ The re-analysis of GEMS data using TAQMan array technology offers real-time detection by molecular techniques for a minimum of 48 targets per sample. While TAQMan is a quick and relatively easy tool to screen for enteric pathogens, disadvantages include cost of equipment and the individual diagnostic cards, training of personnel, laboratory set-up, and the need/ability to manage and analyze large quantities of data [12], [13]. In Mali, The Gambia, and Kenya, studies are underway using TAQMan to assess the impact of rotavirus vaccine on rotavirus disease burden and diarrheal disease etiologies.

Drs. George Armah, Noguchi Memorial Institute, Ghana and Nicola Page, National Institute for Communicable Diseases, South Africa, presented information on the etiology of severe diarrhea based on data from the Regional Reference Laboratories, using TAQMan technology. In West and Central Africa, isolating more than one potential pathogen commonly occurs in children with severe diarrhea. The most prevalent dual infections involved rotavirus, adenovirus, enteroaggregative *E. coli* (EAEC), and enteropathogenic *E. coli* (EPEC). *Shigella* was most frequently associated with rotavirus enzyme immunoassay (EIA)-negative stools. The dominant rotavirus strains in circulation were G1P[8] and G12P[8]. In South and East Africa, Dr. Page discussed using the TAQMan array for more routine surveillance. Many genotypes were in circulation in Mauritius, Rwanda, Zambia, Zimbabwe, and South Africa, including G9P[8], G2P[4], G2P[6], G1P[8], and G8P[6]. Rotavirus remained the top pathogen causing severe diarrhea in all of the countries assessed, except for South Africa, where vaccines were introduced in 2009. While great diversity of rotavirus strains in Africa exists, to date currently used rotavirus vaccines have demonstrated broad cross-protection [1], [14].

Presentations by Dr. Janet Mans, University of Pretoria, South Africa and Dr. Martin Antonio, Medical Research Council Unit, The Gambia, explored the epidemiology of norovirus in Africa. Norovirus, a common cause of traveler’s diarrhea in high resource settings, has increased in relative importance in the US and elsewhere as rotavirus diarrhea has declined [15]. However, there are few large studies describing the burden, epidemiology, and natural history in low resource settings. Due to substantial variation in pathogens based on geography, diarrhea severity, and season, additional research is needed in Africa. The ARN provides a valuable platform that can be leveraged for norovirus surveillance.

### 2.3 Rotavirus vaccine effectiveness and impact

The effectiveness and impact of rotavirus vaccines are currently being assessed at sites across Africa. During this session, representatives from four countries – Botswana, Tanzania, Zambia, and Togo – reported on the experience after introduction of Rotarix® and representatives from Burkino Faso reported on the impact of RotaTeq®. While each study has limited power, in aggregate the data support the early impact of rotavirus vaccines in reducing morbidity and mortality.

In July 2012, the Ministry of Health introduced Rotarix® into the national immunization program in Botswana and established a post-rotavirus vaccine surveillance program. Dr. Margaret Bafana, National Health Laboratory, reported that gastroenteritis-related hospitalizations for children under five decreased by 33 percent during the rainy season post-introduction. There was 54 percent protection with 2 doses and 48 percent protection with 1 dose for children under 6 months of age [16].

Dr. Bavin Jani, WHO, reported data from Tanzania where Rotarix® was introduced in late 2012. After vaccine introduction, confirmed rotavirus cases decreased in infants less than 1 year of age by 50 and 70 percent respectfully, in 2014 and 2015 [17].

Rotavirus surveillance has been on-going in Lusaka, Zambia since 2009 and Rotarix® was introduced in November 2013. Dr. Evans Mpabalwani, University Teaching Hospital, Zambia, reported a 40 percent positivity rate for rotavirus in children admitted to University Teaching Hospital for all causes of diarrhea prior to 2013; numbers declined to 27 percent in 2015 in children under 5 years of age. The number of deaths in children under 1 due to diarrhea dropped from 109 in 2012 to 42 in 2015. These data support the positive impact of rotavirus vaccine, with the largest reductions seen in children under one year of age.

In Togo, where Rotarix® was introduced in 2014, early evidence likewise supports rapid and marked reductions in hospitalizations in the first year post-introduction. As reported by Enyonam Tsolenyanu, Ministry of Health Togo, rotavirus hospitalizations decreased 27 percent during the first post-vaccine year and 46 percent in the second post-vaccine year in children under 5. In children 0 to 11 months, rotavirus hospitalization decreased from 42 percent in the first year after vaccine introduction and 54 percent in the second year after vaccine introduction.

In Burkina Faso, RotaTeq® was introduced in 2013. According to unpublished data presented by Isidore Bonkoungou, National Public Health Laboratory, diarrhea hospitalizations due to rotavirus decreased from 46 to 41 percent in the first post-vaccine year and to 23 percent in the second post-vaccine year in children under 5. The number of diarrhea cases positive for rotavirus fell from 49 to 43 percent in the first post-vaccine year and to 20 percent in the year after vaccine introduction among infants under 1. The proportion of total hospital admissions due to diarrhea decreased from approximately 45 percent pre-vaccine to 22 percent post-introduction.

### 2.4 Post-introduction monitoring of intussusception in African countries

In the US and Europe, there is a low level risk of intussusception with the current licensed rotavirus vaccines, Rotarix® and RotaTeq®. The incidence of intussusception, which occurs naturally in infants, begins to increase at 2–3 months of age and peaks at 4–8 months of age [18], coinciding with the administration of rotavirus vaccines. Thus, temporal associations with vaccines are not necessarily causal. This session contained presentations on intussusception surveillance and monitoring, including country-specific perspectives.

Dr. Evans Mpabalwani, University Teaching Hospital, Zambia provided an overview of intussusception in sub-Saharan Africa based on a review of published data for children under two years of age. In Africa, radiologic confirmation of intussusception is less common than in other parts of the world. Surgery is the mainstay of treatment in sub-Saharan Africa with 77 percent of cases leading to surgical intervention as compared to 28 percent in North America and 20 percent in Europe [19]. Mortality is also highest in sub-Saharan Africa compared to other regions, perhaps due to the late clinical presentation, delays in diagnostic capabilities or undifferentiated diagnosis with diarrheal illness, and lack of medical expertise outside tertiary centers. Since infants die outside the hospital, intussusception is likely under-reported in sub-Saharan Africa [20].

**Fig. 3:**
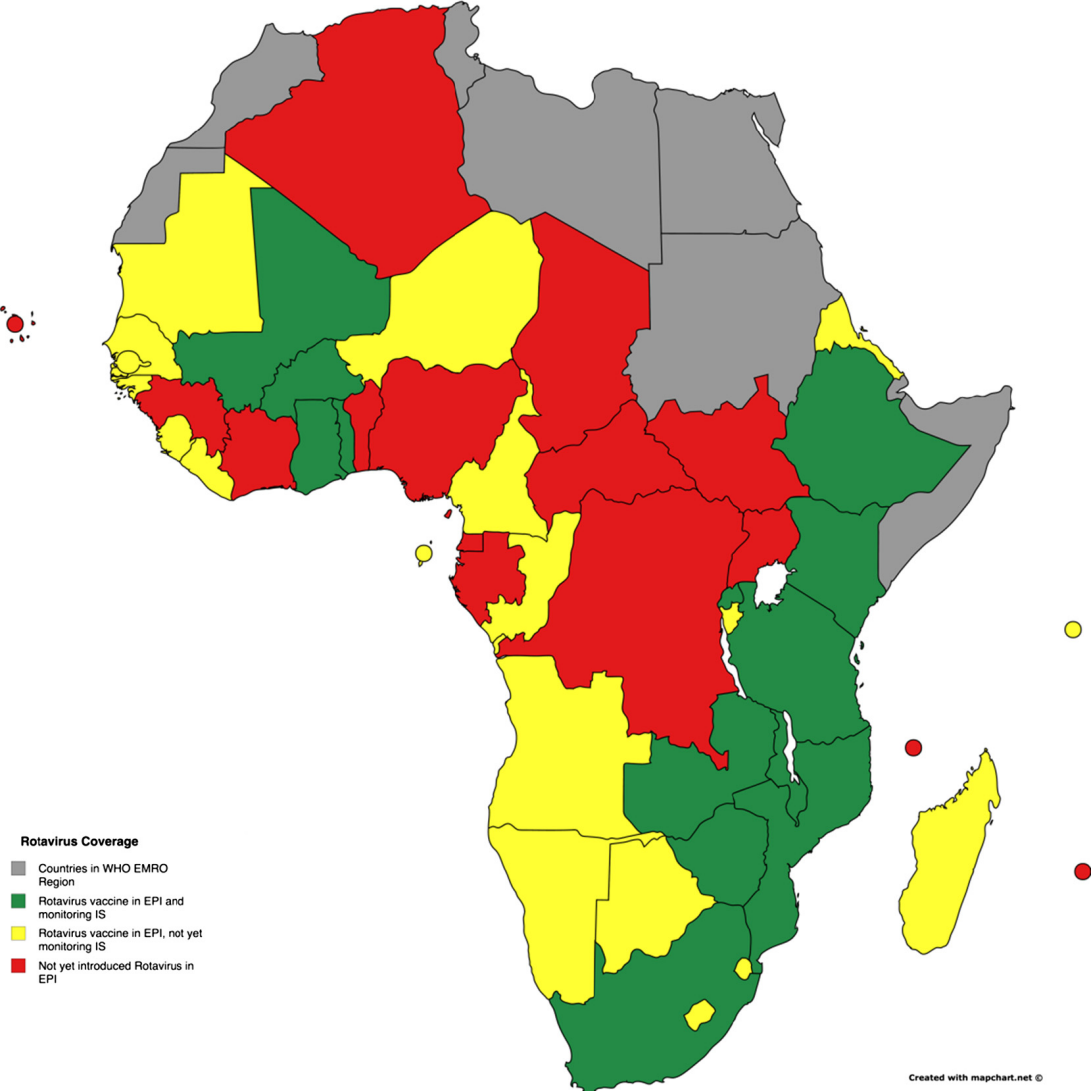
Rotavirus vaccine introduction and intussusception surveillance in African countries. (Global Health Strategies. 2016. Rotavirus vaccine introduction and intussusception surveillance in African countries. Derived from “Review of Naturally Occurring Intussusception in Young Children in the WHO African Region prior to the Era of Rotavirus Vaccine Utilization in the Expanded Programme of Immunization” available online at: http://tropej.oxfordjournals.org/content/early/2016/10/01/tropej.fmw069.full.pdf+html.)

The WHO recommends rotavirus vaccines be given as soon as possible after six weeks of age [21]. However, realizing that children may present late for vaccination, WHO relaxed the age of administration of the first dose of the vaccine beyond 15 weeks based on data from Mexico and Brazil and extrapolation from models suggesting the benefits of rotavirus vaccine outweigh the risk of intussusception [21]. AFRO countries implementing intussusception surveillance to monitor the post-licensure safety of rotavirus vaccines are shown in Fig. 3. It is essential that African countries educate health care workers at all levels about the clinical presentation of intussusception and a potential association with rotavirus vaccine administration during a short “high-risk” window of approximately one to seven days after the first dose [21].

There are limited pre-rotavirus vaccine intussusception data from Africa. Dr. Richard Omore (Kenya), Dr. Joel Bikoroti (Rwanda), Dr. Hope Glover Addy (Ghana), Adama Keita (Mali), and Dr. Tapsoba W. Tousaint (Burkina Faso) provided preliminary unpublished data from ongoing intussusception surveillance programs in their respective countries. While it is too early to evaluate an association between intussusception and rotavirus vaccines in these countries, there are lessons learned for countries beginning surveillance. Monitoring intussusception in sub-Saharan Africa is challenging due to late presentation, delayed diagnosis, death occurring outside the hospital, and under-reporting. Surveillance of intussusception is recommended by WHO as part of safety monitoring of rotavirus vaccines and efforts should continue to improve the recognition and treatment of this serious naturally occurring event.

Because rotavirus vaccines were introduced in 2009, intussusception data are more robust in South Africa than other African countries. Dr. Michele Groome, University of the Witwatersrand, South Africa, reported on progress for an on-going case-control study in South Africa. A total of 263 cases were enrolled in active intussusception surveillance conducted at 8 large pediatric hospitals post-rotavirus vaccine introduction between July 2012 and April 2016; the median age was 6 months and 91 percent of the cases were under 12 months of age. The most common clinical findings at presentation were vomiting and blood in stools; most of the cases were diagnosed by ultrasound. Results evaluating any causal association with rotavirus vaccines are anticipated by the end of 2017.

### 2.5 Short oral presentations/young advocates for infectious disease surveillance in Africa

The ARN established the African Rotavirus Symposium as a mechanism to increase rotavirus awareness in Africa; one objective was to train young researchers and clinicians. The symposium provides a venue to network and share experiences across organizations and countries. This session was specifically for up-and-coming rotavirus advocates to share their work. Among dozens of scientists, clinicians, and public health officials who submitted abstracts, five were selected for oral presentations, of which four are summarized below. The fifth, Dr. Adama Keita (Mali), was included in the intussusception section above.

Dr. Chinedu Chukwubike of Nigeria reported on a 4-year hospital-based surveillance study conducted among children 0 to 59 months, with diarrhea. Over 1600 stool samples were collected from 2011 through 2014 of which 49 percent were positive for rotavirus. The most prevalent genotype combination in the study was G12P[8]. The results highlight the diversity of rotavirus strains in Nigeria prior to vaccine introduction and support the need for continuous strain surveillance, in coordination with vaccine effectiveness evaluations, as part of vaccine introduction.

Belinda Lartey explored the potential association between histo-blood group antigens (HBGAs) and rotavirus infection in a cross-sectional study in Ghanaian children younger than five years. HBGAs may be key receptors for virion attachment and host entry. The HBGAs expressed on the epithelium of the intestines are largely controlled by the FUT2 (secretor) gene that encodes the FUT2 enzyme. The presence or absence of a functional FUT2 enzyme is referred to as an individual’s secretor status and is thought to determine susceptibility or resistance to rotavirus infection. Dr. Lartey’s research shows a significant association between secretor status and rotavirus infection in Ghanaian children under five. Of the rotavirus P-genotypes detected, P[8], P[4], and P[6], P[4] and P[8] were more commonly associated with secretors. Secretors were three times more likely to be infected with rotavirus than non-secretors. These results may have implications for oral rotavirus vaccine response.

In 2010, the Kenya Medical Research Institute/CDC began population-based surveillance to monitor rotavirus in Kenya. Dr. Sammy Khagayi reported that from 2010 to 2013, 25–27 percent of children admitted with acute gastroenteritis were positive for rotavirus compared to post-introduction percentages of 16 and 14 in 2014 and 2015, respectfully. Surveillance data support decreased prevalence since 2014 that is attributable to health care improvements and vaccine introduction. Rotavirus remains an important cause of diarrhea, particularly in children six to eleven months.

Almaz Abebe Tadesse of Ethiopia reported on trends of diarrheal admissions before and after rotavirus vaccine introduction in Ethiopia. In Ethiopia, a total of 145,160 diarrhea cases were documented for 3 years prior to vaccine introduction (2011–2013) and 70,028 cases for 2.5 years post-introduction (2014–2016), with the highest prevalence in children aged 9–12 months. After rotavirus vaccine introduction in November 2013, significant declines in diarrheal hospitalizations and deaths were observed in children less than five years of age with the largest reductions in children under one year, similar to what has been reported in other countries [20].

### 2.6 Advances in rotavirus science: informing public health

The lower immunogenicity of rotavirus vaccines in poor countries is not completely understood. Speakers explored several possibilities for this phenomenon and presented data on the relationship between immunization schedule and dose, gut biome, maternal factors, strain diversity, immunogenicity, whole genome sequencing (WGS) and alternative schedules and doses.

Dr. George Armah, Noguchi Memorial Institute, Ghana, explained how differences in the gut microbiome may impact vaccine efficacy. The high enteropathogenic burden from multiple co-infections changes the intestinal microbiota, which can alter immune response to oral enteric vaccines, like rotavirus. Rotavirus vaccine response negatively correlated with increased bacilli, in particular *Streptococcus bovis*. These findings suggest microbiota may influence rotavirus vaccine response and altering intestinal microbiota could improve immunogenicity.

Dr. Roma Chilengi, Centre for Infectious Disease Research, Zambia, reported on maternal immunity. High maternal IgG titres to rotavirus are present during the vaccination period in infancy, at about six to 32 weeks of age. Human milk mucin binds to rotavirus and inhibits its replication in a dose-dependent manner. Environmental enteric dysfunction (EED) is a syndrome of mucosal and sub-mucosal inflammation that reduces intestinal absorptive capacity and barrier function. EED is a possible explanation for poor vaccine effectiveness in developing countries [22].

Mapaseka Seheri, Rotavirus Reference Laboratory, South Africa, stated studies on rotavirus strain diversity in Africa before and after vaccine introduction show constant evolution and novel strains [14]. Between 2010 and 2015, G1P[8] (21.8 percent), G9P[8] (13.6 percent), G2P[4] (11.5 percent), G12P[8] (6.6 percent), G2P[6] (5.2 percent), and G3P[6] (3.8 percent) were identified as the most common causes of acute rotavirus diarrhea in children under 5. An estimated 42 percent of the strains are regionally important. There is a wide diversity of strain distribution from country to country. Preliminary data from six African countries indicate no clear evidence of strain replacement after vaccine introduction. The findings confirm the common strains worldwide are not present at the same rates in Africa, which is important for evaluating vaccine effectiveness and composition.

Martin Nyaga, University of Free State, South Africa, presented on WGS of rotavirus strain analysis. The diversity of rotavirus strains in the Africa region is generally agreed upon [23], [24], [25] and could be due to the high prevalence of mixed infections with multiple rotavirus strains. None of the genomes of the African strains were vaccine-derived. The unusual genome constellations, co-circulating strains, and reassortants could only be explained by the completeness of WGS. These data support continued surveillance using WGS to monitor the emergence of novel or replacement strains as vaccination programs expand in Africa.

Dr. Fatima Haidara, CVD-Mali, presented research from Mali on a booster dose of rotavirus vaccine at nine months of age to improve the modest efficacy of the vaccine in African countries. Justification for a rotavirus vaccine booster includes weak primary antibody responses, waning protection, and a continued high burden of disease in the second rotavirus season. A nine-month booster dose in Mali did not interfere with measles vaccine immunogenicity and increased rotavirus-specific IgA and IgG levels compared to a placebo. Further research is needed to determine if an extra dose of vaccine at nine months of age could maximize the public health benefit while minimizing delivery cost.

### 2.7 Sustainability and control of diarrhea in African Children: looking to the future

Sustainability, including cost effectiveness, and introduction of rotavirus vaccines into the remaining 21 African countries are continued concerns. This session summarized some of the major challenges for rotavirus vaccines, including: Supply, cold chain storage, cost and cost-effectiveness, efficacy in low-income settings, and optimizing the dose/schedule.

Rotavirus vaccine funding for many African countries relies heavily on Gavi (Fig. 2). Dr. Kathleen Neuzil, CVD, summarized the Gavi model and details of eligibility, transition, and co-financing (http://www.gavi.org/). Deciding which rotavirus vaccine to introduce has implications beyond price that include delivery method, wastage rate, and supply chain. Transition planning is essential: Once a vaccine is introduced into the EPI, countries must sustain the vaccines post-transition and maintain coverage.

Charles Sigei, Public Health Consultant, Kenya evaluated the cost-effectiveness of introducing rotavirus vaccine into Kenya’s national routine immunization program by estimating the health impact and cost of introduction. The model estimated vaccine introduction would save more than US $29 Million in health service costs over 20 birth cohorts. In Kenya, the introduction of rotavirus vaccine was found to be highly cost-effective.

Megan Carey, Bill & Melinda Gates Foundation, stated an important goal of the enteric and diarrheal disease team is to ensure an adequate supply and acceptable presentation of prequalified rotavirus vaccines for delivery to Gavi-eligible countries and lower-middle income countries. In addition to the WHO pre-qualified Rotarix® and Rotateq® vaccines, Rotavac (human live attenuated G9P[11], India), Rotavin (human live attenuated G1P[8], Vietnam), and Lanzhou Lamb (lamb live attenuated G10P[12]) are approved nationally. Several additional oral vaccines, based on human live attenuated and reassortant strains, are in development, as are non-replicating rotavirus vaccines. Ideally, additional vaccines entering the market will alleviate supply concerns and drive prices down.

Goitom Weldegebriel, WHO, summarized integrated approaches for the prevention and control of diarrhea and pneumonia. He gave a broad overview of the Global Action Plan for Pneumonia and Diarrhea (GAPPD), a general framework that provides guidance and coordination to countries and partners for scaling up interventions. Vaccines are just one option to prevent and control diarrhea, but do not protect against all causes of diarrhea; thus, a multi-pronged approach is needed. GAPPD proposes action steps and activities to efficiently move forward and build a broad coalition of global and national policy-makers, planners, donor agencies, and civil society. The full report is available at: http://www.who.int/maternal_child_adolescent/documents/global_action_plan_pneumonia_diarrhoea/en/.

Joseph Biey, WHO, provided an overview of GAPPD in West Africa. Since 1990, the number and rate of under-five births have fallen by more than half. Sub-Saharan Africa had the highest under-five mortality rate in 2015. He concluded immunization though a cost-effective intervention needs to be integrated with other child survival interventions.

## 3 Summary

Rotavirus is the most common cause of severe dehydrating diarrhea among young children in Africa. The past several years have seen unprecedented introduction of rotavirus vaccines into African countries. At this symposium, many countries reported on the impact of rotavirus vaccines in reducing diarrheal morbidity. Intussusception surveillance in Africa is in the early stages. The research agenda is robust. Dr. Jason Mwenda, WHO/AFRO, reaffirmed the importance of the African Rotavirus Surveillance Network to estimate the burden of rotavirus diarrhea in children under five, document rotavirus strains in the region, support awareness and regional advocacy efforts for vaccine introduction, conduct post-marketing surveillance, and evaluate the impact and effectiveness of vaccines.

Priorities include:

• Ensuring the remaining 21 African countries introduce rotavirus vaccine into their EPI, particularly large countries with high burden, such as Nigeria and the Democratic Republic of the Congo.
• Sustaining sentinel surveillance.
• Continuing ongoing and new rotavirus vaccine impact evaluation/effectiveness studies.
• Completing intussusception evaluations and transitioning monitoring and reporting to Ministries of Health
• Monitoring vaccine coverage and improving reporting of coverage and quality of data.

**Fig. 4:**
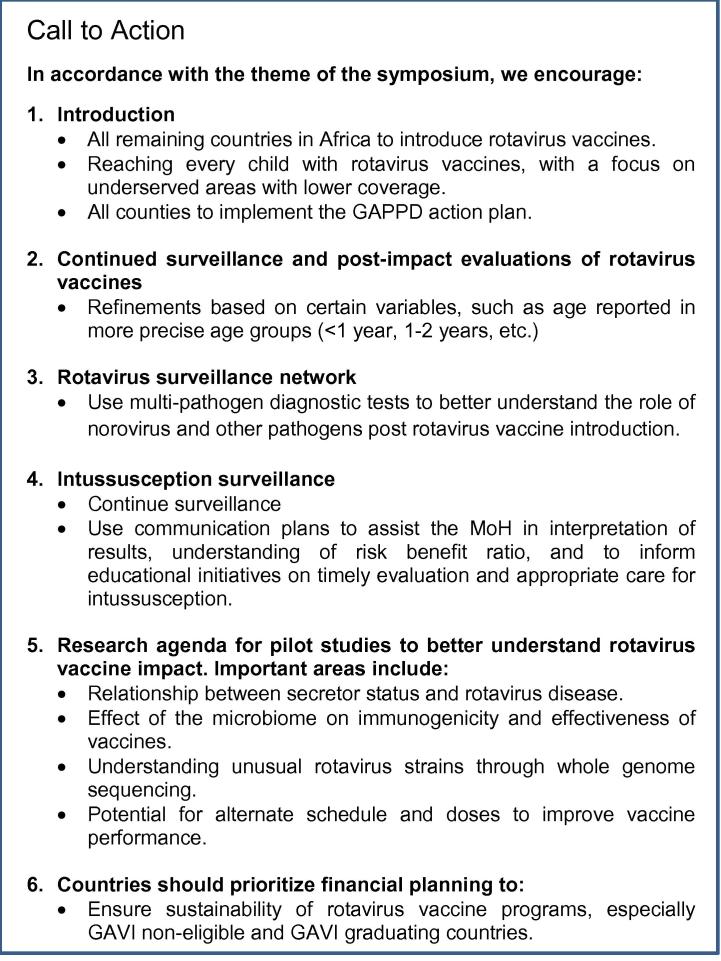
Call to action.

To conclude the symposium, organizers and attendees issued a Call to Action to ensure that every child is reached with rotavirus vaccines (Fig. 4).

## Footnotes

1 The ARN is a network of global rotavirus researchers, African Ministries of Health, the WHO, and key partners such as GAVI, the Vaccine Alliance, the Bill & Melinda Gates Foundation, the pharmaceutical industry, and other stakeholders. The ARN was established in 1998 to support, collaborate, and advance rotavirus research in Africa.

2 The 9th African Rotavirus Symposium concluded the regional rotavirus surveillance network has been critical to documenting burden and epidemiology of rotavirus, assessing seasonal trends, and determining rotavirus genotypes [2].
